# Heavy metal concentrations in Chinese chicken eggs: insights from comparative study of urban and mining areas

**DOI:** 10.7717/peerj.20896

**Published:** 2026-03-11

**Authors:** Xue Li, Yuchen An, Zhuhong Wang

**Affiliations:** 1 School of Public Health, Guizhou Medical University, Guiyang, China; 2 Institute of Surface-Earth System Science, Tianjin University, Tianjin, China

**Keywords:** Heavy metal, Free-range eggs, Commercial eggs, Health risk assessment

## Abstract

**Background:**

The consumption of eggs in China accounts for over 40% of global egg consumption. Since eggs are a crucial source of daily dietary protein, and are relatively accessible and affordable protein foods, the issue of their food safety needs to be taken seriously. Research in some countries has reported excessively high heavy metal levels in eggs from areas surrounding mining sites. However, the extent of heavy metal contamination in eggs from mining regions in China remains poorly understood.

**Methods:**

The concentrations of heavy metals (chromium (Cr), manganese (Mn), cobalt (Co), nickel (Ni), copper (Cu), zinc (Zn), arsenic (As), selenium (Se), cadmium (Cd), and lead (Pb)) in eggs from five cities and three mining areas across China were determined by Inductively coupled plasma mass spectrometry (ICP-MS). Subsequently, the metal concentration was used to evaluate the degree of heavy metal pollution in local eggs, while the estimated daily intake (EDI), target hazard quotient (THQ), hazard index (HI), and carcinogenic risk (CR) were applied to assess the potential health risks of egg consumption.

**Results:**

The results showed that there were significant differences in the concentrations of heavy metals in eggs from different regions, but the overall concentrations (*e.g.*, Cd < 0.05 mg/kg, Pb < 0.02 mg/kg) all complied with China’s national food safety standards for eggs. Within the same region, free-range eggs contained higher heavy metal levels than commercial eggs. The heavy metal HIs of eggs in mining areas were higher than those in non-mining areas, and the HIs of eggs in heavy metal polluted areas were higher than those in non-polluted areas, which made free-range eggs relatively riskier than commercial eggs. This study indicates that free-range eggs may pose a higher risk of heavy metal exposure. In the vast rural areas of China, the potential risk of heavy metal pollution caused by free-range egg farming deserves greater attention.

## Introduction

Eggs are a vital component of the global diet and are rich in high-quality protein. In China, per capita egg consumption is approximately 13.5 kg/year (CNBSC, 2024), accounting for about 40% of total global egg consumption (OECD/FAO, 2022). *The Chinese Dietary Guidelines* recommend one egg per person per day for individuals over 2 years old (Chinese Nutrition Society, 2022). In recent decades, food safety has become a global public health priority, as it directly affects human health and socioeconomic stability (King et al., 2017). As one of the most accessible and nutritionally valuable dietary protein sources, eggs are widely consumed worldwide (Garrido-Miguel et al., 2022). Thus, egg safety has emerged as a critical issue in food quality management. Commercial eggs can be roughly divided into two types based on production systems and feeding methods: free-range eggs and intensive-farmed commercial eggs (Esposito et al., 2016; Voica et al., 2023). Free-range eggs are produced by hens raised in open environments, where hens forage naturally on grass seeds, insects, grains and other native food sources. In contrast, commercial eggs are produced under intensive farming systems, where hens are given standardized synthetic feeds in controlled, artificial rearing environments to improve production efficiency. Elevated heavy metal levels in free-range eggs have been reported in several countries globally. In Australia, researchers found that lead (Pb) concentrations in home-grown eggs from backyard flocks were higher than those in commercial eggs (Grace & MacFarlane, 2016). In Bangladesh, differences in trace elements were observed between free-range eggs and commercial eggs: specifically, copper (Cu), Pb, iron (Fe), and zinc (Zn) contents in free-range eggs exceeding respective standard limits (Samad et al., 2023). In Thailand, poultry near a gold mine were exposed to mercury (Hg), Pb, and cadmium (Cd), and the lifetime cancer risk from Pb and Cd *via* consumption of these contaminated eggs exceeded cancer risk thresholds (Aendo et al., 2022). In China, heavy metal pollution has been reported in free-range duck eggs from the Wuchuan Mercury Mine (Guo et al., 2024). Poultry primarily accumulate heavy metals from feed and the surrounding environment (*e.g.*, soil and water). A portion of these metals can be transferred to eggs, which may pose potential health risks to humans when consumed (Eissa & Shehata, 2024; Panda et al., 2023).

Currently, approximately 460 million people in China live in rural areas, where free-range eggs are one of the main sources of protein for rural residents. However, with China’s rapid economic development, extensive mining activities have exposed many rural residents to elevated mining-related heavy metal pollution risks. Human activities (*e.g.*, mining, chemical industries, vehicular emissions) have released increasing amounts of heavy metals into the environment, which has intensified human heavy metal exposure (Stojić et al., 2023; Zhang et al., 2019). Recent research shows that unregulated metal mining causes heavy metal contamination of rivers, threatening downstream ecosystems and human communities (Macklin et al., 2023). Previous studies have documented excessive heavy metals in surface soils of mining-area villages, endangering local crops and human health (Cai et al., 2023; Liu et al., 2022; Lu et al., 2021). Globally, 14%–17% of farmland is affected by toxic metal pollution, with an estimated 0.9 to 1.4 billion people living in regions facing heightened public health and ecological risks (Hou et al., 2025). Heavy metals pose risks to animals and humans through exposure routes such as ingestion and dermal absorption (Islam et al., 2015; Zhang, Eto & Aikawa, 2021). Upon dietary intake, these metals accumulate in bones or fat and impair the body’s immune function (Brown et al., 2020; Li et al., 2022). Several heavy metals such as chromium (Cr), arsenic (As), Pb, and cadmium (Cd) are categorized as potential carcinogenic metals. Exposure to high levels of As, Cr, and nickel (Ni) could cause skin lesions, neurological and reproductive complications, and cardiovascular diseases (Furtak et al., 2022; Kuo et al., 2021; Zeng et al., 2021). Essential trace elements such as Cu and Zn can maintain the normal physiological operation of the body when consumed in appropriate amounts, but excessive or insufficient intake can cause health problems (Escobedo-Monge et al., 2023). Although prior studies have investigated heavy metals in commercial (Aendo et al., 2022; Zhao et al., 2021) and free-range eggs (Zergui, Boudalia & Joseph, 2023), most of these works focused on individual egg types rather than systematic direct comparisons.

However, in China, research on the differences in heavy metal concentrations between free-range eggs and commercial eggs remains relatively limited. Studies on heavy metal pollution in eggs from mining and other polluted regions are particularly scarce. This research gap carries significant public health implications. Approximately 460 million rural residents depend on free-range eggs as a primary protein source, making the investigation of associated heavy metal contamination particularly urgent and critical. In this study, we measured the concentrations of 10 heavy metals (Cr, Mn, Co, Ni, Cu, Zn, As, Se, Cd, and Pb) in eggs from five cities and three mining areas in China. The objectives of this work are as follows: (1) characterize the heavy metal contamination in free-range and commercial eggs across different regions; (2) compare heavy metal content among eggs from various regions and production systems; (3) evaluate the potential health risks of consuming these eggs for vulnerable populations.

## Materials & Methods

### Study area

It is reported that the main areas of heavy metal pollution in China are concentrated in areas with rapid industrial development, high population density, and frequent mining activities (Duan et al., 2016). We selected typical cities with distinct pollution characteristics as study areas. Shijiazhuang (SJZ) was chosen for its industrial pollution stemming from activities such as electric power production, steel smelting, high-temperature cement calcination, winter heating, chromium salt production, the electroplating industry, and leather production (Yang et al., 2023). Dongguan (DG) was selected due to industrial pollution involving the manufacturing of electroplating, papermaking, electronic component manufacturing, and organic solvent production. Guangzhou (GZ) was included to represent a city with combined industrial (*e.g.*, electrical appliance manufacturing, automobile manufacturing, and chemical industry) and traffic pollution (Liang et al., 2019). Xianyang (XY), a city in Shaanxi Province, was selected mainly due to its industry (such as mining and metallurgy) and agriculture. These sectors exert pressure on the environment, particularly the atmosphere (Wang et al., 2017). Guiyang (GY), China’s first national forest city, is famous for its ecological environment and holiday tourism (Zhao et al., 2022). It was selected as an unpolluted/clean background area in this study. Additionally, three mining regions were designated as study sites: two mercury-mining regions in Wuchuan (WC) and Danzhai (DZ) and one coal-mining region in Emin (EM).

### Sampling and pre-treatment

**Figure 1:**
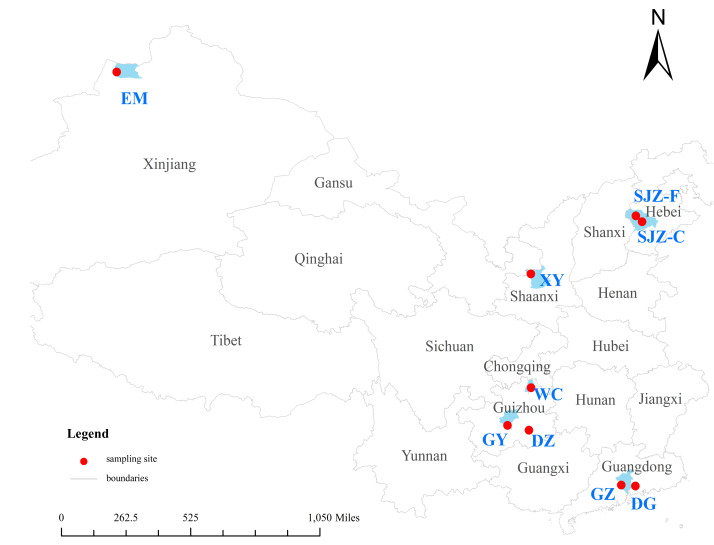
Distribution of egg sampling sites in China.

In this work, a total of 90 chicken eggs were collected. Specifically, 70 free-range eggs were obtained from local poultry farms in four cities (SJZ: *n* = 10; DG: *n* = 10; XY: *n* = 10; GY: *n* = 10) and three mining areas (WC: *n* = 10; DZ: *n* = 10; EM: *n* = 10), while 20 commercial chicken eggs were purchased from local supermarkets in two cities (SJZ: *n* = 10, GZ: *n* = 10) (Fig. 1 and Table 1). At the poultry farms, the hens were fed with locally sourced corn and vegetables. All eggs were labeled and stored at 4 °C in the refrigerator.

In the laboratory, the eggs were washed with Milli-Q water. The washed eggs were air-dried and broken to collect the egg yolk and egg white. The egg yolk and egg white were mixed and combined as a sample in a Polytetrafluoroethylene (PTFE) tube and freeze-dried for 48 h. Each sample (0.50 g, accurately weighed) was digested with five mL of ultrapure HNO_3_ and one mL of ultrapure H_2_O_2_ at 160 °C for 8 h. After cooling to room temperature, the inner chamber of the digestion tank was removed and placed on a hot plate at 90 °C, to evaporate most of the ultrapure water. Finally, the sample volume was fixed to 10 mL with 2% HNO_3_ and stored at 4 °C before measurement.

**Table 1:** Distribution of egg sampling sites in China.

Number	Address	Latitude (N)	Longitude (E)	Date	Sampling area
1	Yuhua District, Shijiazhuang City, Hebei Province	38.006453	114.531362	2024.07	Non-mining Area
2	Xingtang County, Shijiazhuang City, Hebei Province	38.342778	114.165556	2024.07	Non-mining Area
3	Xiegang Town, Dongguan City, Guangdong Province	22.972083	114.141456	2024.08	Non-mining Area
4	Panyu District, Guangzhou City, Guangdong Province	23.024411	113.30828	2024.08	Non-mining Area
5	Emin County, Tacheng Prefecture, Xinjiang Uygur Autonomous Region	46.524673	83.628303	2024.08	Mining Area
6	Xinmin Town, Chenzhou City, Shaanxi Province	35.036389	107.992222	2024.07	Non-mining Area
7	Huaxi District, Guiyang City, Guizhou Province	26.420910	106.619171	2024.07	Non-mining Area
8	Wuchuan County, Zunyi City, Guizhou Province	28.561733	107.999778	2024.07	Mining Area
9	Danzhai County, Qiandongnan Miao and Dong Autonomous Prefecture, Guizhou Province	26.143111	107.876772	2024.07	Mining Area

### Heavy metal analysis

Heavy metals (Cr, Mn, Co, Ni, Cu, Zn, As, Se, Cd, and Pb) were determined using Inductively coupled plasma-mass spectrometry (ICP-MS, ThermoFisher Scientific iCAP RQ; Thermo Fisher Scientific, Waltham, MA, USA) at Guizhou University. Standard reference materials (VAR-CAL-2 for trace elements; CLMS-1 for rare earth elements) were regularly measured to ensure the analytical quality of trace elements by analyzing blank samples, duplicate samples, and reference materials. Standard solutions were prepared using a 10-element standard solution. Calibration curves for the 10 elements were constructed based on six concentration points, and linearity was verified by analyzing these six-gradient concentration. For all 10 elements, the correlation coefficient of the regression line exceeded 0.99. The limits of detection (LOD) and quantification (LOQ) were determined by measuring ten blank samples. To check the accuracy of the analytical method, spike recovery experiments were performed using a certified reference material (CLMS-1) to confirm the absence of heavy metals losses, sample contamination during sample preparation, and matrix interferences during instrumental measurement step. The relative standard deviation (RSD) of the metal was below 10%, and the recoveries of heavy metals were between 80% and 110%, meeting the requirements for trace element analysis in food samples.

### Assessment of human health risk

Health risk assessments were conducted for male adults, female adults, and children.

#### Estimated daily intake

The estimated daily intake (EDI) of heavy metals *via* egg consumption is determined by integrating the average metal concentration in eggs and egg consumption rates among adults and children. The non-carcinogenic risk (NCR) of consuming eggs contaminated with an individual heavy metal is calculated using the target hazard quotient (THQ), while and hazard index (HI) (the sum of the THQ values) is used to evaluate NCR from combine exposure to multiple heavy metals. THQ represents the ratio of actual exposure to the reference oral dose (RfD) (Yi, Yang & Zhang, 2011). HI quantifies cumulative NCR from multiple heavy metals by summing the THQ of each metal (Yin et al., 2021). The corresponding formulas are as follows:

(1) \begin{eqnarray*}\mathrm{EDI}= \frac{{\mathrm{C}}_{\mathrm{i}}\times \mathrm{FIR}}{\mathrm{BW}} \end{eqnarray*}

(2) \begin{eqnarray*}\mathrm{THQ}= \frac{ \left( \mathrm{EF}\times \mathrm{ED}\times \mathrm{FIR}\times {\mathrm{C}}_{\mathrm{i}} \right) }{ \left( \mathrm{RfD}\times \mathrm{BW}\times \mathrm{AT} \right) } \end{eqnarray*}

(3) \begin{eqnarray*}\mathrm{HI}=\sum \mathrm{TH}{\mathrm{Q}}_{\mathrm{i}}\end{eqnarray*}

where EDI is the estimated daily intake of metals (mg/kg/day), C_i_ is the concentration of metals in eggs (mg/kg), FIR is the dietary intake (g/person/day), and BW is body average weight (kg) (U.S. Environmental Protection Agency, 2011). The exposure parameters are shown in Table S1. Where EF is the exposure frequency (d/a), ED is exposure time (a), AT is the average exposure time (d), and BW is the body weight (kg). Exposure parameters are shown in Table S1 and RfD values for target heavy metals are listed in Table S2. The risk threshold criteria are as follows: a THQ or HI value < 1 indicates NCR from heavy metal exposure *via* egg consumption, while values exceeding 1 imply potential adverse non-carcinogenic health effects (U.S. Environmental Protection Agency, 1989).

In addition to NCR, exposure to carcinogenic metals can also induce carcinogenic risk (CR) and total carcinogenic risk (TCR). The CR value of a carcinogen reflects the incremental probability that a consumer will develop cancer over their lifetime due to chronic exposure to that carcinogen. TCR is the sum of the CR values of different metals. The cancer risk from Cr, Cd, and Pb *via* egg consumption was calculated using Formula (4) (U.S. Environmental Protection Agency, 1989).

(4) \begin{eqnarray*}\mathrm{CR}=\mathrm{EDI}\times \mathrm{SF}\end{eqnarray*}

(5) \begin{eqnarray*}\mathrm{TCR}={\mathrm{CR}}_{\mathrm{Cr}}+{\mathrm{CR}}_{\mathrm{Cd}}+{\mathrm{CR}}_{\mathrm{Pb}}\end{eqnarray*}

where EDI (estimated daily intake, mg/kg/day) derived from Formula (2), and SF (cancer slope factor), represents the carcinogenic risk per unit lifetime average daily dose (Aendo et al., 2019). According to the US Environmental Protection Agency (USEPA) guidelines (U.S. Environmental Protection Agency, 2011; U.S. Environmental Protection Agency, 1989), CR and TCR are classified as follows: <1 × 10^−^^6^ is considered negligible and can be ignored, 1 ×10^−^^6^–1 × 10^−^^4^ is deemed an acceptable risk level, and >1 × 10^−^^4^ indicates a potential unacceptable carcinogenic risk.

### Monte Carlo simulation method

Traditional risk assessment methods, as described above, typically rely on average, maximum, or minimum values for risk calculations. However, this approach has significant limitations, including ambiguity regarding the conservatism level and a failure to quantify the uncertainty associated with the final risk estimates. To conduct a more objective risk assessment, this study employs Monte Carlo simulation method (MCS) to characterize the probability distribution of heavy metal intake *via* egg consumptions. Risk assessment models were constructed using the Crystal Ball software (version 11.1.2.4, Oracle, Inc., Nashville, TN, USA). The threshold for assessing endangered exposed populations were defined the as 95th percentile of HI and CR in cumulative probability curves, with 10,000 iterations performed for the simulation (Atamaleki et al., 2020).

### Statistical analysis

ArcGIS Desktop (version 10.8, Esri, Redlands, CA, USA) was used to map the distribution of sampling sites, while SPSS (version 26.0, IBM Corp, Armonk, NY, USA) was used to analyze the data, and Origin Pro 2024 was used to draw graphs. Data were subject to descriptive statistical analysis, with results are expressed as mean values. When the conditions of normality and homogeneity of the variances were observed, the significance of differences in heavy metal concentrations among groups was tested using one-way Analysis of Variance (ANOVA) and Tukey’s honestly significant difference. The significance level was set at *α* = 0.05.

## Results

### Metal concentrations in eggs

**Figure 2:**
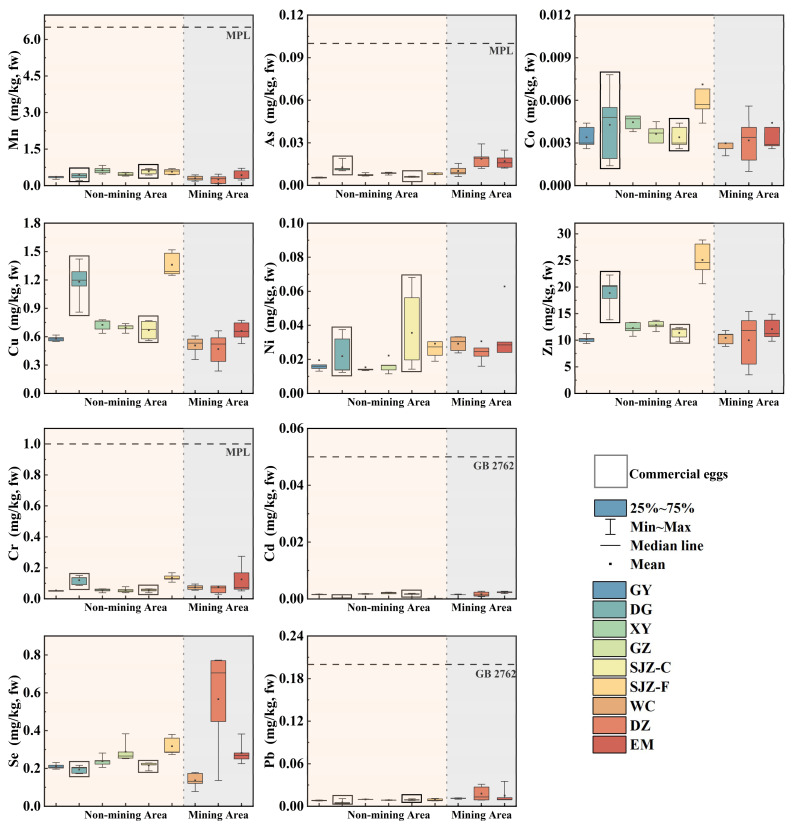
Heavy metal concentrations in eggs from nine areas. Non-mining area: GY, DG, XY, GZ, SJZ-C, SJZ-F; Mining area: WC, DZ, EM. Free-range eggs: GY,DG, XY, SJZ-F,WC, DZ, EM; Commercial eggs: GZ, SJZ-C.

The concentrations of heavy metals (Cr, Mn, Co, Ni, Cu, Zn, As, Se, Cd, and Pb) are shown in Fig. 2. The concentrations of these metals in eggs across eight regions exhibit the following variation ranges, Cr: 0.053–0.14 mg/kg, Mn: 0.25–0.63 mg/kg, Co: 2.3 × 10^−3^–7.1 × 10^−3^ mg/kg, Ni: 0.015–0.063 mg/kg, Cu: 0.47–1.38 mg/kg, Zn: 9.99–25.09 mg/kg, As: 6.0 × 10^−3^–0.019 mg/kg, Pb: 5.4 × 10^−3^–0.018 mg/kg, Se: 0.14–0.57, Cd: 1.5 ×1 0^−3^–2.3 × 10^−3^ mg/kg.

In our study, significant regional differences are observed in the levels of Mn, Co, Cu, As, Se, Cd, and Pb in eggs (*P* < 0.05). Eggs collected from GZ, XY, SJZ, GY, and WC exhibited a consistent order of metal concentration: Zn > Cu > Mn > Se > Cr > Ni > Pb > As > Co > Cd. Although heavy metal concentrations vary considerably among eggs across the five cities and three mining areas, all detected values are below the permissible limit specified in the food safety standards issued by the FAO/WHO, the National Food Safety Standard for Contaminants in Foods (GB 2762-2022), and the European Commission (Table S2). EM eggs have the highest Cr, Ni, and Cd concentrations, while DZ eggs possess the highest As, Se, and Pb levels; both are mining areas. Considering the different feeding methods of free-range eggs and commercial egg production systems, we further compared the heavy metal levels between free-range eggs (sampled from seven regions) and commercial eggs (sampled from GZ and SJZ). Notable differences in heavy metal profiles are identified between these two egg types. Specifically, in SJZ, the concentrations of Cu, Zn, As, Se, and Cd differed significantly between free-range and commercial eggs (*P* < 0.05). Additionally, free-range eggs from DG and commercial eggs from GZ also exhibit distinct concentrations of Cr, Cu, Zn, As, Se, and Cd (*P* < 0.05). In general, the levels of harmful heavy metal elements in free-range eggs (across all 7 sampling regions) are higher than those in the commercial ones (from GZ and SJZ).

### Human health risk assessment

#### Non-carcinogenic risk assessment

**Figure 3:**
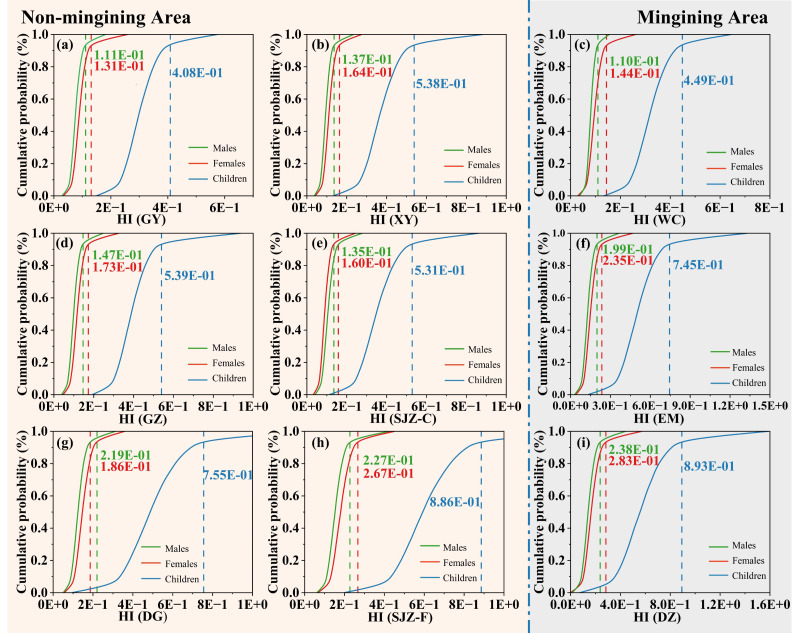
Non-carcinogenic probabilistic health risk assessment for all heavy metals. Free-range eggs: GY, WC, DG, XY, SJZ-F, DZ, EM; Commercial eggs: GZ, SJZ-C.

The calculated EDI values of the heavy metals are provided in Table S3. The highest EDIs for males, females, and children are associated with Zn from DG (0.010, 0.012, and 0.039). Conversely, the lowest THQs for males, females, and children are link to Cd in eggs from DG (1.1 × 10^−7^, 1.3 × 10^−7^, and 4.2 × 10^−7^). The EDI values of all 10 heavy metals *via* egg consumption are below the RfD for all three population groups (children, males, and females). In contrast, the highest THQs for males, females, and children are associated with Se in eggs from DZ (0.123, 0.148, and 0.476). The lowest THQs are alternatively recorded for Cd in eggs from XY (1.6 × 10^−4^, 2.6 × 10^−4^, and 8.6 × 10^−3^) (Table S4). According to the MCS, the HI values at the 95th percentile for males, females, and children are shown in Fig. 3 and Table S4. The maximum HI values *via* egg consumption are 0.238, 0.283, and 0.893 for males, females, and children, respectively. All HI values at the 95th percentile are below the threshold of 1, indicating an absence of non-carcinogenic health risks.

#### Carcinogenic risk assessment

**Figure 4:**
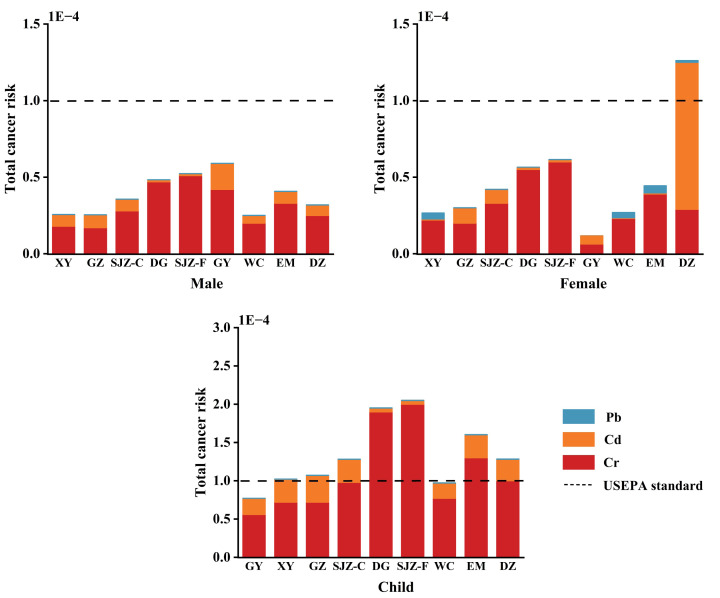
Total carcinogenic risk assessment of heavy metals at the 95th percentile by using MCS in eggs from nine areas. Free-range eggs: GY, WC, DG, XY, SJZ-F, DZ, EM; Commercial eggs: GZ, SJZ-C.

Table 2 presents the CR values of Cr, Cd, and Pb for males, females, and children based on the MCS. For males, the highest estimated CRs of Cr, Cd, and Pb from egg consumption are 5.1 × 10^−5^, 1.7 × 10^−5^, and 1.0 × 10^−7^, respectively. For females, the highest estimated CRs of Cr, Cd, and Pb from egg intake are 6.0 × 10^−5^, 9.6 × 10^−5^, and 4.7 × 10^−6^, respectively. For children, the highest estimated CRs of Cr, Cd, and Pb from egg ingestion are 2.0 × 10^−4^, 3.5 × 10^−5^, and 4.2 × 10^−7^, respectively. Notably, for children, CRs of Cr in free-range eggs from four areas (DG, SJZ, DZ, and EM) exceed the threshold of 1 × 10^−4^ for the child population. Figure 4 presents the TCR for males, females, and children based on the MCS. For males, the highest estimated TCR is 5.9 × 10^−5^, observed in GY. For females, the highest estimated TCR is 1.3 × 10^−5^ in DZ. For children, estimated TCRs from all areas except GY and WC exceed the threshold of 1 × 10^−4^.

**Table 2:** Carcinogenic risk assessment of heavy metals at the 95th percentile by using MCS in eggs from nine areas.

Area	Cr			Cd			Pb		
	Males	Females	Children	Males	Females	Children	Males	Females	Children
GY	4.2×10^−5^	6.3×10^−6^	5.6×10^−5^	1.7×10^−5^	5.4×10^−6^	2.1×10^−5^	5.0×10^−8^	1.4×10^−8^	1.7×10^−7^
WC	2.0×10^−5^	2.3×10^−5^	7.7×10^−5^	5.0×10^−6^	4.7×10^−7^	2.0×10^−5^	5.9×10^−8^	3.5×10^−6^	2.3×10^−7^
DG-F	4.7×10^−5^	5.5×10^−5^	**1.9×10** ^−4^	1.3×10^−6^	1.5×10^−6^	5.0×10^−6^	5.0×10^−8^	5.9×10^−8^	2.0×10^−7^
XY	1.8×10^−5^	2.2×10^−5^	7.2×10^−5^	7.6×10^−6^	7.1×10^−7^	3.0×10^−5^	5.8×10^−8^	3.9×10^−6^	2.3×10^−7^
GZ-C	1.7×10^−5^	2.0×10^−5^	7.2×10^−5^	8.4×10^−6^	1.0×10^−5^	3.5×10^−5^	4.9×10^−8^	5.8×10^−8^	2.0×10^−7^
SJZ-C	2.8×10^−5^	3.3×10^−5^	9.8×10^−5^	7.6×10^−6^	9.0×10^−6^	3.0×10^−5^	5.4×10^−8^	6.4×10^−8^	2.1×10^−7^
SJZ-F	5.1 ×10^−5^	6.0 ×10^−5^	**2.0×10** ^−4^	1.3×10^−6^	1.5×10^−6^	4.9×10^−6^	5.9×10^−8^	7.0×10^−8^	2.4×10^−7^
DZ	2.5×10^−5^	2.9×10^−5^	**1.0×10** ^−4^	6.8×10^−6^	9.6×10^−5^	2.8×10^−5^	1.0×10^−7^	1.2×10^−6^	4.2×10^−7^
EM	3.3×10^−5^	3.9×10^−5^	**1.3×10** ^−4^	7.7×10^−6^	7.2×10^−7^	3.0×10^−5^	7.9×10^−8^	4.7×10^−6^	3.1×10^−7^

**Notes.**

CR ≥1.0 ×10^−4^ may have a potential health risk (indicated in the bold). Free-range eggs: GY, WC, DG-F, XY, SJZ-F, DZ, EM; Commercial eggs: GZ-C, SJZ-C.

## Discussion

### Analysis of heavy metal pollution

Our results reveal distinct differences in heavy metal concentrations between free-range and commercial eggs within the same region, which can be attributed to their contrasting production modes and exposure pathways. For free-range hens, heavy metals in soil—especially in mining areas—accumulate in local crops, weeds, and insects (Zhuang, Zou & Shu, 2009). Hens ingest these contaminants during foraging, or absorbed them through contaminated drinking water. This exposure pathway is highly dependent on the local environmental background. For example, in mining-influenced areas, soil concentrations of Cd and Pb are often elevated (Cai et al., 2023), leading to higher potential accumulation in free-range eggs.

By contrast, commercial hens are reared in standardized facilities with strict environmental and dietary controls. Their feed comes from commercial suppliers that comply with China’s Feed Hygiene Standard (GB 13078-2017), which limits heavy metal content in feed. Drinking water is also treated to reduce contamination risks. Moreover, before being sold in supermarkets, commercial eggs must undergo mandatory food safety testing. Tests include the analysis heavy metals (*e.g.*, for Pb, Cd, and As) in line with the National Food Safety Standard for Contaminants in Foods (GB 2762-2022). This full-chain supervision—from feed production to post-harvest testing—explains two key points: first, commercial eggs have lower heavy metal concentrations; second, their contamination sources are fewer: primarily restricted to feed and water, rather than soil or wild forage.

**Figure 5:**
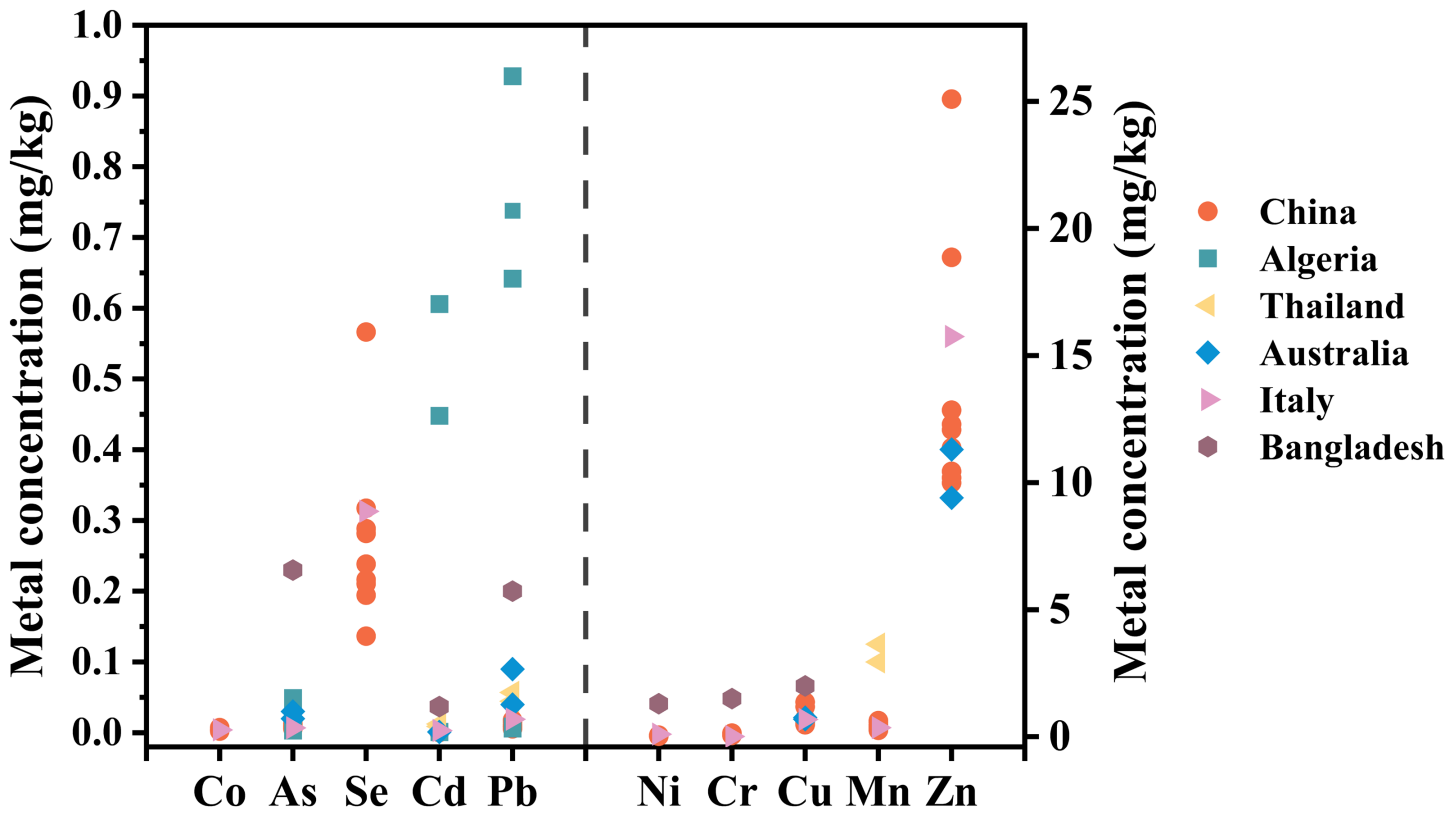
Heavy metal concentrations in eggs from different areas (mg/kg).

To contextualize our findings globally, we compared our data with heavy metal levels in eggs reported from other regions (Fig. 5). Notably, concentrations of toxic heavy metals (Pb, Cd, and As) in our study’s eggs—including those from mining areas—are significantly lower than those documented in heavily polluted regions. For example, the mean Pb concentrations in our samples is 0.012 mg/kg, which is lower than that in eggs from Thai gold mining areas (Aendo et al., 2022; mean: 0.06–0.12 mg/kg), as well as those in eggs from contaminated sites in Italy (Esposito et al., 2016) and Bangladesh (Islam et al., 2015). This discrepancy may be linked to China’s stricter feed heavy metal regulations and the low transfer efficiency of dietary heavy metals to eggs: previous studies have shown that most ingested heavy metals (*e.g.*, 99% of Pb and Cd) are excreted in hen feces, with only a small fraction (<1%) transferred to eggs (Heuel et al., 2023; Wang et al., 2023; Wang et al., 2024). For commercial hens, this low transfer rate is further mitigated by low heavy metal levels in controlled feed, while for free-range hens in non-mining areas, the relatively clean soil background reduces initial exposure. These two factors collectively contribute to the low heavy metal levels observed in our samples.

### Health risk of heavy metals to local residents

Heavy metals in eggs can be ingested by humans and accumulate in human organs and tissues, thereby posing potential health risks to consumers. Therefore, a targeted health risk assessment is essential to this study.

As, Cd, Hg, and Pb have been classified as potentially toxic elements in the human diet since 1972 (World Health Organization, 1972). EDI is determined by analyzing the average metal concentration in eggs and the daily egg consumption rates of adults and children (Varol et al., 2022). In the present study, the EDIs of all the 10 heavy metals from eggs are less than the RfD, for children, males, and females. Monte Carlo simulated (MCS) method, recommended by the US Environmental Protection Agency (USEPA), is employed in this study to improve, the precision of risk assessment by accounting for inherent uncertainties. The estimation of non-carcinogenic health hazards due to the consumption of metal-contaminated food is conducted using the THQ, one of the most authoritative methods for such assessments. The average cumulative health risk (HI) is calculated by summing the THQ of the 10 heavy metals. According to the USEPA, the acceptable value of THQ is 1. The HI values of free-range eggs in GY, XY, and WC are similar to those of commercial eggs in GZ and SJZ. In SJZ, the HI of free-range eggs is higher than that of commercial eggs; the reverse pattern is observed in GZ. Overall, free-range eggs exhibit distinct HI profiles compared to commercial eggs within the same region. To sum up, the NCRs of heavy metals for males, females, and children are less than 1, indicating no significant health risks associated with consuming individual heavy metals or a mixture through egg ingestion. Previous studies reported similar or lower for NCR values. These results are in accordance with those reported in Algeria unpolluted areas (Zergui, Boudalia & Joseph, 2023). Notably, children have higher NCRs than adults. Further comparative analysis reveals that in the same area, free-range eggs generally have higher HI values than commercial eggs—this trend is more obvious in mining-influenced areas.

In addition to non-carcinogenic risks, the carcinogenic potential of heavy metals *via* egg consumption also requires attention. Among these metals, Cr, Cd, and Pb are classified as probable human carcinogens. Environmental pollutant exposure is a well-documented risk factor for cancer—though cancer can be caused by multiple factors (*e.g.*, age, genetics, and bad habits), exposure to such pollutants increases the risk (Furtak et al., 2022). In the dietary context, Cr, Cd, and Pb are regarded as potentially toxic elements. Thus, this study specifically assesses the carcinogenic risk of Cr, Cd, and Pb (probable human carcinogens) in eggs. The CR values of all the eggs in this study are lower than that of free-range eggs near abandoned gold mines in Thailand (Aendo et al., 2022). And these results are in accordance with those reported in Bangladesh (Samad et al., 2023). Across all groups, CR and TCR is highest in children, followed by females, and then males. Among the three elements, the CR follows the order: Cr > Cd > Pb. Importantly, Cr is identified as the primary contributor to CR, highlighting its role as the dominant carcinogenic metal in the four areas (DG, SJZ, DZ, and EM) (Table 2). The TCR values simulated by MCS showed that long-term and continuous consumption of eggs with heavy metal levels below the regulatory limits still poses potential carcinogenic risks to most children. Therefore, when selecting free-range eggs, consumers should pay attention to the production areas.

## Conclusions

This study determined the concentrations of 10 heavy metals (Cr, Mn, Co, Ni, Cu, Zn, As, Se, Cd, Pb) in eggs collected from nine areas and evaluated the associated health risks. Results showed that all egg samples meet the current national safety standards. However, free-range eggs—especially those from mining areas (*e.g.*, SJZ, DG)—exhibit higher heavy metal concentrations than commercial eggs. Specifically, the concentration of Cd, Pb, and Cr in free-range eggs from mining-area are 1.5 to 3.5 times higher than commercial or free-range eggs from non-mining. Additionally, children have 1.8 to 2.2 times higher NCR than adults. This makes free-range eggs relatively riskier than commercial eggs. Among these metals, Cr accounted for 60% to 75% of the TCR. Considering heavy metal bioaccumulation and daily egg consumption, targeted risk mitigation measures are recommended: (1) Local authorities should prioritize quarterly monitoring of Cr, Cd, Pb in free-range eggs from mining and ecologically vulnerable areas; (2) Free-range farmers should be guided to avoid foraging near mining waste and use feed with low-heavy-metal content.

This study is limited by its 9-region sample (with a lack of representation from southern rural areas) and exclusively focus on egg-derived risks (excluding other dietary, and non-dietary exposures). Future research should expand the sampling scope, integrate multi-source dietary data, and explore the link between soil and egg heavy metal concentrations. This will provide scientific support for optimizing national free-range egg safety standards.

## Supplemental Information

10.7717/peerj.20896/supp-1 Supplemental Information 1 Supplemental Tables

## Additional Information and Declarations

### Data Availability

The following information was supplied regarding data availability: The raw measurements are available in the Supplementary File.
