# Supplementary material for: Heavy metal concentrations in Chinese chicken eggs: insights from comparative study of urban and mining areas
Source: PeerJ. 2026 Mar 11;14:e20896. doi: 10.7717/peerj.20896 (PMC12988732; doi:10.7717/peerj.20896)
Supplement: Supplemental Information 1 [file peerj-14-20896-s001.docx]

**Table S1. Parameters and values of heavy metal exposures.**

| **Exposure parameters** | **Unit** | **Males** | **Females** | **Children** |
| --- | --- | --- | --- | --- |
| AT | d | 10950 | 10950 | 3650 |
| BW ^1,2^ | kg | 69.6 | 59.0 | 16.0 |
| EF | d/a | 365 | 365 | 365 |
| ED | a | 30 | 30 | 10 |
| FIR ^1,2^ | g/person/day | 37.0 | 37.0 | 33.3 |

**Table S2. Heavy metal concentrations for evaluation parameters and food safety standards.**

|  | **Cr** | **Mn** | **Co** | **Ni** | **Cu** | **Zn** | **As** | **Se** | **Cd** | **Pb** |
| --- | --- | --- | --- | --- | --- | --- | --- | --- | --- | --- |
| RfD (mg/kg/day) ^3^ | 3×10^-3^ | 0.14 | 3.0×10^-4^ | 0.02 | 0.04 | 0.3 | 3.0×10^-4^ | 5.0×10^-3^ | 1.0×10^-3^ | 3.5×10^-3^ |
| SF ^4^ | 0.5 | - | - | 0.91 | - | - | 1.5 | - | 6.3 | 8.5×10^-3^ |
| GB 2762-2022 ^5^ | - | - | - | - | - | - | - | - | 0.05 | 0.2 |
| MPL (mg/kg/day) | 1.0 ^6^ | 6.5 ^7^ | - | - | - | - | 0.1 ^8^ | - | 0.1 ^8^ | 0.1 ^8^ |

**Table S3. Estimated daily intake of heavy metals in eggs from 9 areas.**

| **Area** | **EDI (mg/kg)** | | | | | | | | | |
| --- | --- | --- | --- | --- | --- | --- | --- | --- | --- | --- |
|  | **Cr** | **Mn** | **Co** | **Ni** | **Cu** | **Zn** | **As** | **Se** | **Cd** | **Pb** |
| **Males** | |  |  |  |  |  |  |  |  |  |
| GY | 2.8×10^-5^ | 1.9×10^-4^ | 1.2×10^-6^ | 1.0×10^-5^ | 3.1×10^-4^ | 5.4×10^-3^ | 2.9×10^-6^ | 1.1×10^-4^ | 8.5×10^-7^ | 4.3×10^-6^ |
| WC | 3.9×10^-5^ | 1.6×10^-4^ | 1.6×10^-6^ | 1.6×10^-5^ | 2.7×10^-4^ | 5.5×10^-3^ | 5.4×10^-6^ | 7.3×10^-5^ | 8.0×10^-7^ | 5.9×10^-6^ |
| DG | 6.4×10^-5^ | 2.5×10^-4^ | 2.3×10^-6^ | 1.2×10^-5^ | 6.3×10^-4^ | 1.0×10^-2^ | 6.8×10^-6^ | 1.0×10^-4^ | 1.1×10^-7^ | 2.9×10^-6^ |
| XY | 2.9×10^-5^ | 3.3×10^-4^ | 2.4×10^-6^ | 8.2×10^-6^ | 3.9×10^-4^ | 6.5×10^-3^ | 4.0×10^-6^ | 1.3×10^-4^ | 9.6×10^-7^ | 5.2×10^-6^ |
| GZ | 2.9×10^-5^ | 2.5×10^-4^ | 1.9×10^-6^ | 1.2×10^-5^ | 3.7×10^-4^ | 6.8×10^-3^ | 4.6×10^-6^ | 1.5×10^-4^ | 1.1×10^-6^ | 4.6×10^-6^ |
| SJZ-C | 4.4×10^-5^ | 3.1×10^-4^ | 1.8×10^-6^ | 1.9×10^-5^ | 3.6×10^-4^ | 6.0×10^-3^ | 3.2×10^-6^ | 1.2×10^-4^ | 9.6×10^-7^ | 4.8×10^-6^ |
| SJZ-F | 7.2×10^-5^ | 3.0×10^-4^ | 2.3×10^-6^ | 1.6×10^-5^ | 7.2×10^-4^ | 1.3×10^-2^ | 4.4×10^-6^ | 1.7×10^-4^ | 1.1×10^-7^ | 4.8×10^-6^ |
| DZ | 4.1×10^-5^ | 1.4×10^-4^ | 1.7×10^-6^ | 1.6×10^-5^ | 2.5×10^-4^ | 5.3×10^-3^ | 9.9×10^-6^ | 3.0×10^-4^ | 9.0×10^-7^ | 9.5×10^-6^ |
| EM | 6.7×10^-5^ | 2.3×10^-4^ | 2.3×10^-6^ | 3.3×10^-5^ | 3.5×10^-4^ | 6.4×10^-3^ | 9.0×10^-6^ | 1.5×10^-4^ | 1.2×10^-6^ | 8.0×10^-6^ |
| **Females** | |  |  |  |  |  |  |  |  |  |
| GY | 3.3×10^-5^ | 2.3×10^-4^ | 1.4×10^-6^ | 1.2×10^-5^ | 3.6×10^-4^ | 6.4×10^-3^ | 3.4×10^-6^ | 1.3×10^-4^ | 1.0×10^-6^ | 5.1×10^-6^ |
| WC | 4.6×10^-5^ | 1.9×10^-4^ | 1.9×10^-6^ | 1.8×10^-5^ | 3.2×10^-4^ | 6.5×10^-3^ | 6.3×10^-6^ | 8.6×10^-5^ | 9.4×10^-7^ | 7.0×10^-6^ |
| DG | 7.5×10^-5^ | 2.8×10^-4^ | 2.7×10^-6^ | 1.4×10^-5^ | 7.4×10^-4^ | 1.2×10^-2^ | 8.0×10^-6^ | 1.2×10^-4^ | 1.3×10^-7^ | 3.4×10^-6^ |
| XY | 3.4×10^-5^ | 3.9×10^-4^ | 2.8×10^-6^ | 9.7×10^-6^ | 4.5×10^-4^ | 7.7×10^-3^ | 4.7×10^-6^ | 1.5×10^-4^ | 1.1×10^-6^ | 6.1×10^-6^ |
| GZ | 3.4×10^-5^ | 3.0×10^-4^ | 2.3×10^-6^ | 1.4×10^-5^ | 4.4×10^-4^ | 8.1×10^-3^ | 5.4×10^-6^ | 1.8×10^-4^ | 1.3×10^-6^ | 5.4×10^-6^ |
| SJZ-C | 5.2×10^-5^ | 3.7×10^-4^ | 2.1×10^-6^ | 2.2×10^-5^ | 4.2×10^-4^ | 7.1×10^-3^ | 3.8×10^-6^ | 1.4×10^-4^ | 1.1×10^-6^ | 5.7×10^-6^ |
| SJZ-F | 8.5×10^-5^ | 3.6×10^-4^ | 4.5×10^-6^ | 1.8×10^-5^ | 8.5×10^-4^ | 1.6×10^-2^ | 5.2×10^-6^ | 2.0×10^-4^ | 1.3×10^-7^ | 5.7×10^-6^ |
| DZ | 4.8×10^-5^ | 1.6×10^-4^ | 2.0×10^-6^ | 1.9×10^-5^ | 2.9×10^-4^ | 6.3×10^-3^ | 1.2×10^-5^ | 3.6×10^-4^ | 1.1×10^-6^ | 1.1×10^-5^ |
| EM | 7.9×10^-5^ | 2.7×10^-4^ | 2.8×10^-6^ | 3.9×10^-5^ | 4.1×10^-4^ | 7.6×10^-3^ | 1.1×10^-5^ | 1.8×10^-4^ | 1.4×10^-6^ | 9.4×10^-6^ |
| **Children** | |  |  |  |  |  |  |  |  |  |
| GY | 1.1×10^-4^ | 7.5×10^-4^ | 4.8×10^-6^ | 4.1×10^-5^ | 1.2×10^-3^ | 2.1×10^-2^ | 1.1×10^-5^ | 4.4×10^-4^ | 3.3×10^-6^ | 1.7×10^-5^ |
| WC | 1.5×10^-4^ | 6.3×10^-4^ | 6.2×10^-6^ | 6.1×10^-5^ | 1.1×10^-3^ | 2.2×10^-2^ | 2.1×10^-5^ | 2.8×10^-4^ | 3.1×10^-6^ | 2.3×10^-5^ |
| DG | 2.5×10^-4^ | 9.4×10^-4^ | 8.9×10^-6^ | 4.6×10^-5^ | 2.5×10^-3^ | 3.9×10^-2^ | 2.7×10^-5^ | 4.4×10^-4^ | 4.2×10^-7^ | 1.1×10^-5^ |
| XY | 1.1×10^-4^ | 1.3×10^-3^ | 9.4×10^-6^ | 3.2×10^-5^ | 1.5×10^-3^ | 2.6×10^-2^ | 1.6×10^-5^ | 5.0×10^-4^ | 3.7×10^-6^ | 2.0×10^-5^ |
| GZ | 1.1×10^-4^ | 9.8×10^-4^ | 7.5×10^-6^ | 4.6×10^-5^ | 1.4×10^-3^ | 2.7×10^-2^ | 1.8×10^-5^ | 6.0×10^-4^ | 4.4×10^-6^ | 1.8×10^-5^ |
| SJZ-C | 1.7×10^-4^ | 1.2×10^-3^ | 7.1×10^-6^ | 7.4×10^-5^ | 1.4×10^-3^ | 2.4×10^-2^ | 1.3×10^-5^ | 4.5×10^-4^ | 3.7×10^-6^ | 1.9×10^-5^ |
| SJZ-F | 2.8×10^-4^ | 1.2×10^-3^ | 1.5×10^-5^ | 6.1×10^-5^ | 2.8×10^-3^ | 5.2×10^-2^ | 1.7×10^-5^ | 6.6×10^-4^ | 4.2×10^-7^ | 1.9×10^-5^ |
| DZ | 1.6×10^-4^ | 5.3×10^-4^ | 6.7×10^-6^ | 6.4×10^-5^ | 9.8×10^-4^ | 2.1×10^-2^ | 3.9×10^-5^ | 1.2×10^-3^ | 3.5×10^-6^ | 3.7×10^-5^ |
| EM | 2.6×10^-4^ | 8.9×10^-4^ | 9.1×10^-6^ | 1.3×10^-4^ | 1.4×10^-3^ | 2.5×10^-2^ | 3.5×10^-5^ | 5.9×10^-4^ | 4.8×10^-6^ | 3.1×10^-5^ |
| **RfD** | 3.0×10^-3^ | 1.4×10^-1^ | 3.0×10^-4^ | 2.0×10^-2^ | 4.0×10^-2^ | 3.0×10^-1^ | 3.0×10^-4^ | 5.0×10^-3^ | 1.0×10^-3^ | 3.5×10^-3^ |

Free-range eggs: GY, WC, DG-F, XY, SJZ-F, DZ, EM. Commercial eggs: GZ-C, SJZ-C.

**Table S4. Target hazard quotient (THQ) and hazard index (HI) of heavy metals in eggs from 9 areas.**

| **Area** | **THQ** | | | | | | | | | | **HI** |
| --- | --- | --- | --- | --- | --- | --- | --- | --- | --- | --- | --- |
|  | **Cr** | **Mn** | **Co** | **Ni** | **Cu** | **Zn** | **As** | **Se** | **Cd** | **Pb** |  |
| **Males** |  |  |  |  |  |  |  |  |  |  |  |
| GY | 0.016 | 2.3×10^-3^ | 6.4×10^-3^ | 1.1×10^-3^ | 0.012 | 0.028 | 0.015 | 0.034 | 1.3×10^-3^ | 1.8×10^-3^ | 0.111 |
| WC | 0.020 | 2.0×10^-3^ | 8.6×10^-3^ | 1.1×10^-3^ | 0.010 | 0.026 | 0.031 | 0.024 | 1.1×10^-3^ | 2.3×10^-3^ | 0.109 |
| DG | 0.036 | 3.6×10^-3^ | 0.017 | 1.3×10^-3^ | 0.025 | 0.053 | 0.039 | 0.032 | 2.2×10^-4^ | 1.8×10^-3^ | 0.186 |
| XY | 0.016 | 4.0×10^-3^ | 0.013 | 6.9×10^-4^ | 0.015 | 0.034 | 0.021 | 0.040 | 1.5×10^-3^ | 2.2×10^-3^ | 0.139 |
| GZ | 0.017 | 2.8×10^-3^ | 0.010 | 1.5×10^-3^ | 0.014 | 0.035 | 0.024 | 0.050 | 1.8×10^-3^ | 2.0×10^-3^ | 0.147 |
| SJZ-C | 0.034 | 3.7×10^-3^ | 0.010 | 2.3×10^-3^ | 0.014 | 0.031 | 0.016 | 0.035 | 1.5×10^-3^ | 2.1×10^-3^ | 0.135 |
| SJZ-F | 0.039 | 3.5×10^-3^ | 0.026 | 1.5×10^-3^ | 0.028 | 0.071 | 0.023 | 0.054 | 1.6×10^-4^ | 2.3×10^-3^ | 0.227 |
| DZ | 0.032 | 2.3×10^-3^ | 0.012 | 1.9×10^-3^ | 0.012 | 0.038 | 0.062 | 0.123 | 1.9×10^-3^ | 6.0×10^-3^ | 0.238 |
| EM | 0.055 | 3.4×10^-3^ | 0.019 | 5.9×10^-3^ | 0.014 | 0.035 | 0.053 | 0.050 | 1.9×10^-3^ | 5.8×10^-3^ | 0.199 |
| **Females** |  |  |  |  |  |  |  |  |  |  |  |
| GY | 0.019 | 2.7×10^-3^ | 7.5×10^-3^ | 1.3×10^-3^ | 0.014 | 0.032 | 0.017 | 0.040 | 1.5×10^-3^ | 2.2×10^-3^ | 0.131 |
| WC | 0.026 | 2.5×10^-3^ | 0.011 | 1.5×10^-3^ | 0.013 | 0.034 | 0.040 | 0.030 | 1.4×10^-3^ | 3.0×10^-3^ | 0.144 |
| DG | 0.043 | 4.2×10^-3^ | 0.020 | 1.5×10^-3^ | 0.030 | 0.063 | 0.047 | 0.037 | 2.6×10^-4^ | 2.2×10^-3^ | 0.219 |
| XY | 0.019 | 4.7×10^-3^ | 0.015 | 8.0×10^-4^ | 0.018 | 0.039 | 0.025 | 0.047 | 1.7×10^-3^ | 2.6×10^-3^ | 0.162 |
| GZ | 0.020 | 3.3×10^-3^ | 0.012 | 1.8×10^-3^ | 0.017 | 0.041 | 0.028 | 0.059 | 2.1×10^-3^ | 2.3×10^-3^ | 0.173 |
| SJZ-C | 0.040 | 4.3×10^-3^ | 0.012 | 2.7×10^-3^ | 0.017 | 0.037 | 0.019 | 0.042 | 1.8×10^-3^ | 2.5×10^-3^ | 0.160 |
| SJZ-F | 0.046 | 4.2×10^-3^ | 0.031 | 1.7×10^-3^ | 0.033 | 0.083 | 0.027 | 0.063 | 2.6×10^-4^ | 2.7×10^-3^ | 0.267 |
| DZ | 0.038 | 2.7×10^-3^ | 0.015 | 2.2×10^-3^ | 0.014 | 0.044 | 0.072 | 0.148 | 2.3×10^-3^ | 7.2×10^-3^ | 0.283 |
| EM | 0.065 | 4.0×10^-3^ | 0.022 | 7.0×10^-3^ | 0.016 | 0.041 | 0.064 | 0.058 | 2.3×10^-3^ | 6.6×10^-3^ | 0.235 |
| **Children** |  |  |  |  |  |  |  |  |  |  |  |
| GY | 0.061 | 8.5×10^-3^ | 0.024 | 4.1×10^-3^ | 0.043 | 0.101 | 0.055 | 0.126 | 4.8×10^-3^ | 6.9×10^-3^ | 0.408 |
| WC | 0.081 | 8.0×10^-3^ | 0.035 | 4.6×10^-3^ | 0.041 | 0.107 | 0.126 | 0.096 | 4.5×10^-3^ | 9.5×10^-3^ | 0.451 |
| DG | 0.140 | 0.014 | 0.067 | 4.9×10^-3^ | 0.180 | 0.212 | 0.154 | 0.124 | 8.7×10^-4^ | 7.1×10^-3^ | 0.755 |
| XY | 0.062 | 0.015 | 0.049 | 2.7×10^-3^ | 0.058 | 0.131 | 0.082 | 0.154 | 5.7×10^-3^ | 8.7×10^-3^ | 0.538 |
| GZ | 0.063 | 0.011 | 0.038 | 5.7×10^-3^ | 0.052 | 0.128 | 0.087 | 0.186 | 6.6×10^-3^ | 7.3×10^-3^ | 0.539 |
| SJZ-C | 0.133 | 0.014 | 0.040 | 8.9×10^-3^ | 0.055 | 0.122 | 0.064 | 0.138 | 5.8×10^-3^ | 8.4×10^-3^ | 0.531 |
| SJZ-F | 0.152 | 0.014 | 0.103 | 5.7×10^-3^ | 0.110 | 0.272 | 0.088 | 0.211 | 8.6×10^-4^ | 8.7×10^-3^ | 0.886 |
| DZ | 0.125 | 8.7×10^-3^ | 0.048 | 7.0×10^-3^ | 0.044 | 0.141 | 0.232 | 0.476 | 7.3×10^-3^ | 0.029 | 0.893 |
| EM | 0.216 | 0.013 | 0.072 | 0.022 | 0.053 | 0.130 | 0.203 | 0.186 | 7.1×10^-3^ | 0.022 | 0.745 |

Free-range eggs: GY, WC, DG-F, XY, SJZ-F, DZ, EM; Commercial eggs: GZ-C, SJZ-C.

**Reference**

(1) Ju, L.; Zhao, L.; Fang, H.; Guo, Q.; Pu, W.; Xu, X.; Li, S.; Chen, X.; Cai, S.; Yu, D. Main food intakes of Chinese children aged 6 – 17 years, 2016 – 2017: surveillance data-based evaluation. *China Journal of Public Health* **2023**, *39*, 550-555.

(2) CNBSC. China Statistical Yearbook. **2024**.

(3) USEPA. Regional Screening Level (RSL) Summary Table; Environmental Protection Agency. **2024**.

(4) USPEA. Rish Assessment Guidance for Guidance for Superfund Volume I: Human Health Evaluation Manual（Part A）；Environmental Protection Agency. **1989**.

(5) Anonymous. China National food safety standard. Limits for contaminants in food. *National Health Commossion of the People's Republic of China* **2022**.

(6) FAO/WHO. Codex Alimentarius—general standards for contaminants and toxins in food. Schedule 1 Maximum and Guideline levels for contaminants and toxins in food. **2010**.

(7) WHO. Environmental health criteria IPCS. International Program of Chemical Society. 1998. https://wiki.potsdam.edu/wikichem/index.php/Environmental_Health_Criteria#:~:text=Environmental%20Health%20Criteria%20%28EHC%29%20is%20a%20series%20of,and%20published%20by%20the%20World%20Health%20Organization%20%28WHO%29.

(8) JECFA. Codex general standard for contaminants and toxins in food and feeds. *In: 64th meeting of the Joint FAO/WHO Expert Committee on Food Additives (JECFA)*. 2005. https://www.fao.org/food-safety/scientific-advice/jecfa/en/ (accessed.
